# Permanent Draft Genome Sequence of *Rhizobium* sp. Strain LCM 4573, a Salt-Tolerant, Nitrogen-Fixing Bacterium Isolated from Senegalese Soils

**DOI:** 10.1128/genomeA.00285-17

**Published:** 2017-05-04

**Authors:** Nathalie Diagne, Erik Swanson, Céline Pesce, Fatoumata Fall, Fatou Diouf, Niokhor Bakhoum, Dioumacor Fall, Mathieu Ndigue Faye, Rediet Oshone, Stephen Simpson, Krystalynne Morris, W. Kelley Thomas, Lionel Moulin, Diegane Diouf, Louis S. Tisa

**Affiliations:** a Centre National de Recherches Agronomiques, Institut Sénégalais de Recherches Agricoles (CNRA/ISRA), Bambey, Senegal; b Laboratoire Mixte International Adaptation des Plantes et Microorganismes Associés aux Stress Environnementaux (LAPSE), Centre de Recherche de Bel Air, Dakar-Bel Air, Senegal; c University of New Hampshire, Durham, New Hampshire, USA; d Laboratoire Commun de Microbiologie IRD/ISRA/UCAD, Centre de Recherche de Bel Air, Dakar, Senegal; e Centre National de Recherches Forestières, Institut Sénégalais de Recherches Agricoles (CNRA/ISRA), Hann Dakar, Senegal; f Institut de Recherche Pour le Développement (IRD), UMR IPME 34394, Montpellier, France; g Département de Biologie Végétale, Université Cheikh Anta Diop (UCAD), Dakar, Senegal

## Abstract

The genus *Rhizobium* contains many species that are able to form nitrogen-fixing nodules on plants of the legume family. Here, we report the 5.5-Mb draft genome sequence of the salt-tolerant *Rhizobium* sp. strain LCM 4573, which has a G+C content of 61.2% and 5,356 candidate protein-encoding genes.

## GENOME ANNOUNCEMENT

Rhizobia are a diverse group of alpha- and betaproteobacteria that form nitrogen-fixing symbiosis with legumes (1, 2). This group currently consists of more than 100 species dispatched in 13 genera (http://www.rhizobia.co.nz). During the past decade, the number of rhizobial species increased dramatically, especially in the genus *Rhizobium* (3). The symbiotic relationship with legumes results in the formation of a special structure on the root of the legume called the nodule (4, 5). Inside the nodule, the bacteria obtain their nutrients from the plant and in exchange produce a reduced form of nitrogen from atmospheric dinitrogen (process of biological nitrogen fixation, or BNF).

Rhizobial host plants belong to the *Leguminosae* family, which is the third largest family of angiosperms. The legume family includes roughly 730 genera and over 19,400 species (6) and is divided into three subfamilies: the *Caesalpinioideae*, *Mimosoideae*, and *Papilionoideae* (7). The rhizobia-legume symbiosis provides several advantages for improving soil fertility and agricultural productivity (8). This symbiosis, by providing nitrogen to plants, limits the requirement of chemical fertilizers and thus groundwater pollution from nitrates (9). Legumes also serve as an alternative source of protein for human and animal consumption (10).

Many members of the genus *Rhizobium* are able to form nodules on a broad range of legumes, while others are very specific. *Rhizobium* sp. strain LCM 4573 was isolated from the rhizosphere of soil around *Prosopis juliflora* under saline conditions in the peanut basin in Senegal (11). The isolate also infects and forms root nodules on *Acacia seyal* plants. Under *in vitro* culture conditions, this strain is able to tolerate up to 700 mM NaCl. Because of these properties, this strain could potentially be used in association with leguminous plants for the reforestation of saline lands. The *Rhizobium* sp. strain LCM 4573 genome was sequenced to provide information on its physiology and ecology and to identify molecular markers that are involved in its tolerance to salinity.

Sequencing of the draft genome of *Rhizobium* sp. strain LCM 4573 was performed at the Hubbard Center for Genome Studies (University of New Hampshire, Durham, NH, USA) using Illumina technology techniques (12). A standard Illumina shotgun library was constructed and sequenced using the Illumina HiSeq2500 platform, which generated 9,210,384 reads (260-bp insert size) totaling 2,219 Mb. The Illumina sequence data were trimmed by Trimmomatic version 0.32 (13) and assembled using SPAdes version 3.5 (14) and ALLPaths-LG version r52488 (15). The final draft assembly for *Rhizobium* sp. strain LCM 4573 consisted of 30 contigs with an *N*_50_ contig size of 378.2.7 kb and 301.9× coverage of the genome. The final assembled genome had a total sequence length of 5,521,535 bp with a G+C content of 61.2%.

The assembled *Rhizobium* sp. strain LCM 4573 genome was annotated via the NCBI Prokaryotic Genome Annotation Pipeline (PGAP), resulting in 5,356 candidate protein-encoding genes, 48 tRNAs, and 2 rRNA regions.

### Accession number(s).

This whole-genome shotgun sequence has been deposited at DDBJ/EMBL/GenBank under the accession number MDDW00000000. The version described in this paper is the first version, MDDW01000000.

## References

[1] Chen WM, Moulin L, Bontemps C, Vandamme P, Béna G, Boivin-Masson C 2003 Legume symbiotic nitrogen fixation by beta-proteobacteria is widespread in nature. J Bacteriol 185:7266–7272. doi:10.1128/JB.185.24.7266-7272.2003. 14645288 PMC296247

[2] MacLean AM, Finan TM, Sadowsky MJ 2007 Genomes of the symbiotic nitrogen-fixing bacteria of legumes. Plant Physiol 144:615–622. doi:10.1104/pp.107.101634. 17556525 PMC1914180

[3] Mousavi SA, Li L, Wei GH, Räsänen L, Lindström K 2016 Evolution and taxonomy of native mesorhizobia nodulating medicinal *Glycyrrhiza* species in China. Syst Appl Microbiol 39:260–265. doi:10.1016/j.syapm.2016.03.009. 27105685

[4] van Berkum P, Terefework Z, Paulin L, Suomalainen S, Lindström K, Eardly BD 2003 Discordant phylogenies within the *rrn* loci of rhizobia. J Bacteriol 185:2988–2998. doi:10.1128/JB.185.10.2988-2998.2003. 12730157 PMC154066

[5] Lin DX, Wang ET, Tang H, Han TX, He YR, Guan SH, Chen WX 2008 *Shinella kummerowiae* sp. nov., a symbiotic bacterium isolated from root nodules of the herbal legume *Kummerowia stipulacea*. Int J Syst Evol Microbiol 58:1409–1413. doi:10.1099/ijs.0.65723-0. 18523187

[6] Shamseldin A, Abdelkhalek A, Sadowsky MJ 2017 Recent changes to the classification of symbiotic, nitrogengen-fixing legume-associating bacteria: a review. Symbiosis 71:91–109. doi:10.1007/s13199-016-0462-3.

[7] Lu YL, Chen WF, Han LL, Wang ET, Chen WX 2009 *Rhizobium alkalisoli* sp. nov., isolated from *Caragana intermedia* growing in saline-alkaline soils in the north of China. Int J Syst Evol Microbiol 59:3006–3011. doi:10.1099/ijs.0.007237-0. 19643906

[8] Cardoso D, Pennington RT, de Queiroz LP, Boatwright JS, Van Wyk B-E, Wojciechowski MF, Lavin M 2013 Reconstructing the deep-branching relationships of the papilionoid legumes. S Afr J Bot 89:58–75. doi:10.1016/j.sajb.2013.05.001.

[9] Vitousek PM, Menge DNL, Reed SC, Cleveland CC 2013 Biological nitrogen fixation: rates, patterns and ecological controls in terrestrial ecosystems. Philos Trans R Soc Lond B Biol Sci 368:20130119. doi:10.1098/rstb.2013.0119. PMC3682739 23713117

[10] Berrada H, Benbrahim KF 2014 Taxonomy of the rhizobia: current prespectives. Br Microbiol Res J 4:616–639. doi:10.9734/BMRJ/2014/5635.

[11] Fall F 2016 Impact de Sporobolus robustus Kunth sur la microflore symbiotique et al.’établissement de légumineuses à usages mutiples dans des sols salés du Delta du Sine—Saloum au Sénégal. PhD thesis Université Cheikh Anta Diop de Dakar, Dakar, Senegal.

[12] Bennett S 2004 Solexa Ltd. Pharmacogenomics 5:433–438. doi:10.1517/14622416.5.4.433. 15165179

[13] Bolger AM, Lohse M, Usadel B 2014 Trimmomatic: a flexible trimmer for Illumina sequence data. Bioinformatics 30:2114–2120. doi:10.1093/bioinformatics/btu170. 24695404 PMC4103590

[14] Nurk S, Bankevich A, Antipov D, Gurevich AA, Korobeynikov A, Lapidus A, Prjibelski AD, Pyshkin A, Sirotkin A, Sirotkin Y, Stepanauskas R, Clingenpeel SR, Woyke T, McLean JS, Lasken R, Tesler G, Alekseyev MA, Pevzner PA 2013 Assembling single-cell genomes and mini-metagenomes from chimeric MDA products. J Comput Biol 20:714–737. doi:10.1089/cmb.2013.0084. 24093227 PMC3791033

[15] Gnerre S, MacCallum I, Przybylski D, Ribeiro FJ, Burton JN, Walker BJ, Sharpe T, Hall G, Shea TP, Sykes S, Berlin AM, Aird D, Costello M, Daza R, Williams L, Nicol R, Gnirke A, Nusbaum C, Lander ES, Jaffe DB 2011 High-quality draft assemblies of mammalian genomes from massively parallel sequence data. Proc Natl Acad Sci U S A 108:1513–1518. doi:10.1073/pnas.1017351108. 21187386 PMC3029755

